# Altered expression of Tumor Necrosis Factor Alpha -Induced Protein 3 correlates with disease severity in Ulcerative Colitis

**DOI:** 10.1038/s41598-017-09796-9

**Published:** 2017-08-25

**Authors:** Ishani Majumdar, Vineet Ahuja, Jaishree Paul

**Affiliations:** 1 School of Life Sciences, Jawaharlal Nehru University, New Delhi, India; 2 Department of Gastroenterology, All India Institute of Medical Sciences, New Delhi, India

## Abstract

Ulcerative colitis (UC), an inflammatory disorder of the colon arises from dysregulated immune response towards gut microbes. Transcription factor NFκB is a major regulatory component influencing mucosal inflammation. We evaluated expression of Tumor Necrosis Factor Alpha Induced Protein3 (TNFAIP3), the inhibitor of NFκB activation and its associated partners ITCH, RNF11 and Tax1BP1 in inflamed mucosa of UC patients. We found highly significant up-regulated mRNA expression of TNFAIP3 that negatively correlated with disease activity in UC. mRNA levels of ITCH, RNF11 and Tax1BP1 were significantly down-regulated. Significant positive correlation with disease activity was noted for Tax1BP1. All four genes showed significant down-regulation at protein level. mRNA levels of inducers of TNFAIP3 expression, NFκB p65 subunit and MAST3 was determined. There was significant increase in p65 mRNA expression and down-regulated MAST3 expression. This suggested that increase in NFκB expression regulates TNFAIP3 levels. Deficiency of TNFAIP3 expression resulted in significant up-regulation of NFκB p65 sub-unit as well as its downstream genes such as iNOS, an inflammatory marker, inhibitors of apoptosis like cIAP2 and XIAP and mediators of anti-apoptotic signals TRAF1 and TRAF2. Taken together, decreased expression of TNFAIP3 and its partners contribute to inflammation and up-regulation of apoptosis inhibitors that may create microenvironment for colorectal cancer.

## Introduction

Inflammatory bowel diseases (IBD) are chronic, relapsing and idiopathic disorders of the gastrointestinal tract that arise from complex interactions between genetic factors of the host, commensal microflora and environmental factors leading to dysregulation of the immune system. Ulcerative colitis (UC) and Crohn’s disease (CD) are two sub-types of IBD differing in few histological and pathological features. IBD occurrence in India tends to follow a north-south divide where UC and CD is predominant in north and south India respectively^1^. Incidence and prevalence rates of UC reported in India are the highest in Asia^2^. Due to its rising incidence rates, current interest in various aspects of IBD like pathological mechanisms, treatment and epidemiology is rapidly growing. UC is characterized by inflammation of the colonic mucosa spreading proximally from rectum to the entire colon. An aberrant immune response arising from intolerance towards luminal antigens and commensal microflora is a hallmark of UC^3^. This dysregulated immune response is mainly mediated by various cytokines responsible for initiation and augmentation of the disease resulting in mucosal injury during UC^4^. Cytokines activate nuclear factor kappa-light-chain-enhancer of activated B cells (NFκB) pathway, induce production of inflammatory mediators and inhibit apoptosis^5^. Increased activation of NFκB is one of the major drivers for dysregulated inflammatory response during the disease^6–8^. NFκB controls the transcription of various genes involved in apoptosis, cell-cycle progression and proliferation^9^. Chronic inflammation in IBD, particularly UC is a predisposing factor for development of colorectal cancer (CRC). IBD associated CRC constitutes approximately 5% of all CRC cases^10^. 20–30% of patients with longstanding IBD (more than 30 years) are at a high risk of developing CRC^10^.

Aberrantly activated NFκB is a key player in pathogenesis of various diseases like atherosclerosis, asthma and diabetes^11–13^. Therefore, regulation of NFκB activation is required to prevent inflammation and damage. The cytoplasmic ubiquitin editing molecule Tumor Necrosis Factor Alpha Induced Protein3 (TNFAIP3, also known as A20) negatively regulates NFκB activation. Expressed at basal levels in resting cells, it is rapidly induced after NFκB activation in response to various stimuli such as tumor necrosis factor alpha (TNFα), interleukin 1 (IL-1), lipopolysaccharide (LPS)^14–16^. It consists of N-terminal deubiquitinating and C-terminal E3 ubiquitin ligase domain thereby facilitating deubiquitination of target proteins like Receptor-interacting serine/threonine-protein kinase 1 (RIP1) and Tumor necrosis factor receptor-associated factor 6 (TRAF6) followed by their ubiquitination and proteasomal degradation^14^. TNFAIP3 interacts with two E3 ligases ITCH and RING finger protein 11 (RNF11) and an adaptor protein Tax1 (Human T-Cell Leukemia Virus Type I) Binding Protein 1 (Tax1BP1) to form the “ubiquitin editing complex” and is dependent on these molecules to inhibit NFκB signaling^17–19^. Absence of any of these complex members prevents binding and action of TNFAIP3 on its target molecules^18^. ITCH mediates binding of TNFAIP3 and Tax1BP1 with substrates^17^. Deficiency of ITCH and RNF11 in mice causes spontaneous inflammation and persistent NFκB activation^18, 20^. Therefore, both these ubiquitin ligases are required by TNFAIP3 to inactivate NFκB signaling^21^. Tax1BP1 is an ubiquitin receptor linking TNFAIP3 with ubiquitinated substrates like RIP1 and TRAF6^22^. Mice lacking Tax1BP1 are susceptible to lethality from low doses of TNFα and IL-1^23^. ITCH, RNF11 and Tax1BP1 are important in preventing chronic inflammation^24–26^. TNFAIP3 also has anti-apoptotic role and regulates tight junction proteins in intestinal epithelial cells^27, 28^.

TNFAIP3 is particularly important to maintain homeostatic NFκB activation and suppress inflammation in the intestinal milieu where mucosal surfaces are constantly exposed to luminal bacteria. It’s deficiency in mice models leads to development of severe intestinal inflammation^27, 28^. TNFAIP3 is identified as a susceptibility gene in development of CD and its expression decreases during moderate to severe CD^29, 30^. Further it has also been identified as one of the biomarkers for poor response to treatment given to CD patients^30^.

Therefore, we hypothesize that differential expression of the ubiquitin editing complex members may play a role in hyper-activation of NFκB during UC. The aim of this study was to determine their expression at mRNA and protein level in inflamed colon and how they influence pathogenesis of the disease. This study reveals that expression of all four members of the ubiquitin editing complex members is altered at both mRNA and protein level which leads to dysregulated expression of some key NFκB regulated genes that are involved in outcome of the disease.

## Results

### mRNA expression of ubiquitin editing complex members is altered during UC and correlates with disease activity

**Figure 1:**
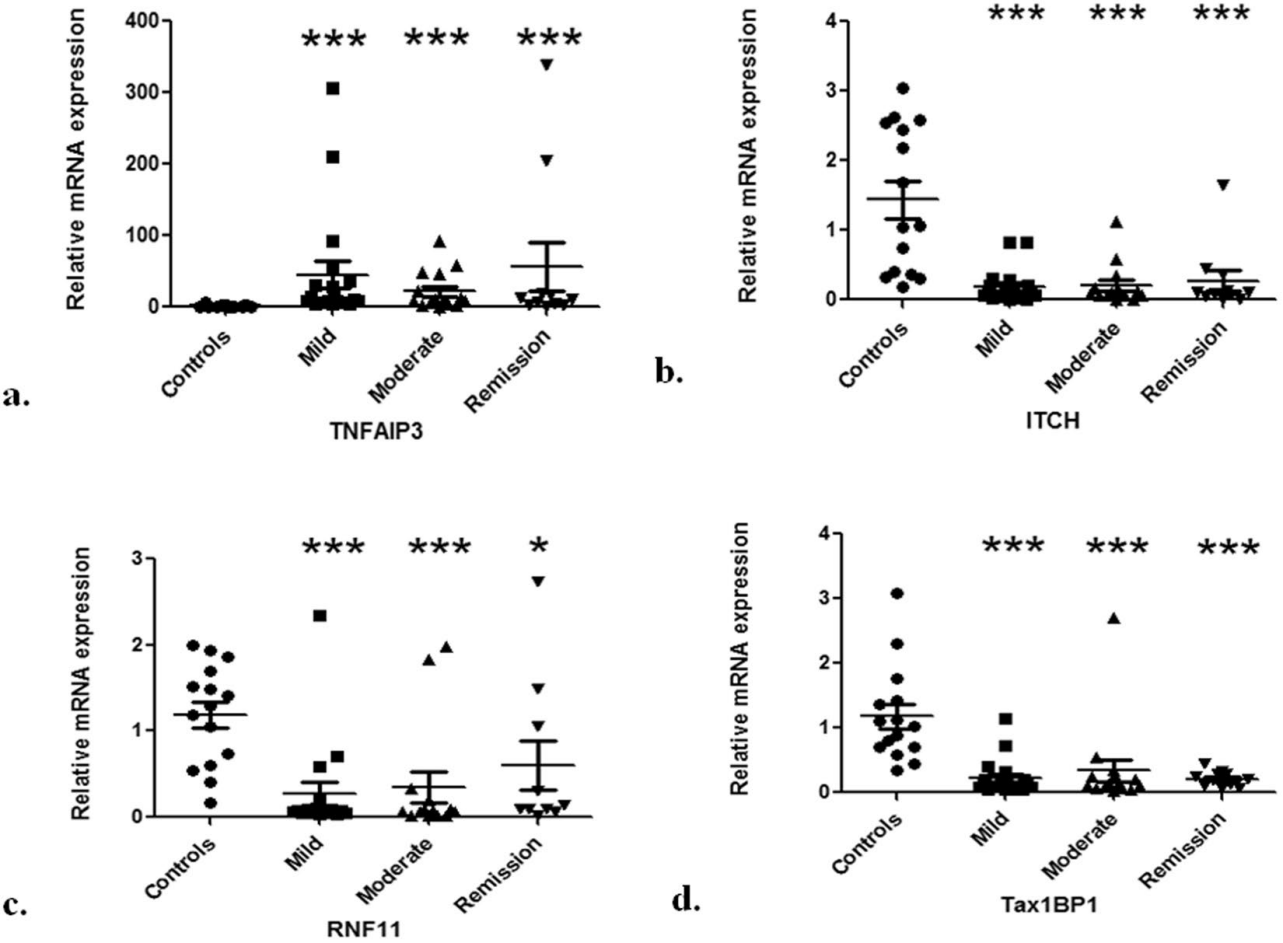
Altered mRNA expression of ubiquitin editing complex members during UC Relative mRNA levels estimated by quantitative real time PCR for (**a**) TNFAIP3 (**b**) ITCH (**c**) RNF11 and (**d**) Tax1BP1 in inflamed colonic mucosal biopsies. Controls (n = 15), mild (n = 19), moderate (n = 13) and remission (n = 14) UC patients. GAPDH was used as the reference gene. Data represented as means ± SEM ***P < 0.001 *P < 0.05 represents statistical significance of difference in expression with controls.

We determined the mRNA expression of members of ubiquitin editing complex i.e. TNFAIP3, ITCH, RNF11 and Tax1BP1 in inflamed colon biopsies from UC patients. Mean fold change in mRNA expression in controls and UC patients and statistical significance indicated by p values between each group are summarized in Table 1. TNFAIP3 mRNA expression was up-regulated in patients. In mild UC patients mRNA expression of TNFAIP3 increased by 45 fold as compared to controls (p < 0.0001). TNFAIP3 mRNA levels were 21.9 fold higher in moderate UC group than controls (p = 0.0003). Interestingly, the TNFAIP3 mRNA showed a trend for lower expression in moderate UC patients as compared to mild UC group (p = 0.41). Whereas in remission patients, TNFAIP3 mRNA expression increased by 56.2 fold with respect to controls (p = 0.0007) (Fig. 1a).

**Table 1:** mRNA levels of ubiquitin editing complex members.

UEC member	Controls	Mild UC	Moderate UC	Remission	Controls vs Mild UC	Controls vs Moderate UC	Controls vs Remission	Mild vs moderate UC	Mild UC vs Remission	Moderate UC vs Remission
	Mean Fold change				P value (Significant p-values are underlined)					
TNFAIP3	1.7	45.0	21.9	56.2	<0.0001	0.0003	0.0007	0.41	0.44	1.00
ITCH	1.4	0.2	0.2	0.3	<0.0001	<0.0001	0.0006	0.88	0.85	0.92
RNF11	1.2	0.3	0.3	0.6	<0.0001	0.0008	0.0184	0.77	0.11	0.14
Tax1BP1	1.2	0.2	0.3	0.2	<0.0001	<0.0001	<0.0001	0.91	0.35	0.53

On the other hand, mRNA expression of associated members ITCH, RNF11 and Tax1BP1 decreased in patients. ITCH mRNA expression decreased to 0.2 fold in both mild and moderate UC groups as compared to controls (both p < 0.0001) (Table 1). Amongst patients in remission, ITCH expression reduced to 0.3 fold with respect to controls (p = 0.0006) (Fig. 1b). While for RNF11 and Tax1BP1, in mild UC patients highly significant decrease in their mRNA levels was noted. RNF11 expression decreased to 0.3 fold whereas Tax1BP1 expression reduced to 0.2 fold (p < 0.0001). In patients with moderate UC, expression of both RNF11 and Tax1BP1 mRNA was decreased to 0.3 fold (p = 0.0008 and p < 0.0001 respectively). In remission patients however, 0.6 fold decrease in RNF11 expression was observed (p = 0.0184) (Fig. 1c). For Tax1BP1 on the other hand, 0.2 fold decrease in mRNA expression was noted (p < 0.0001) (Fig. 1d).

**Figure 2:**
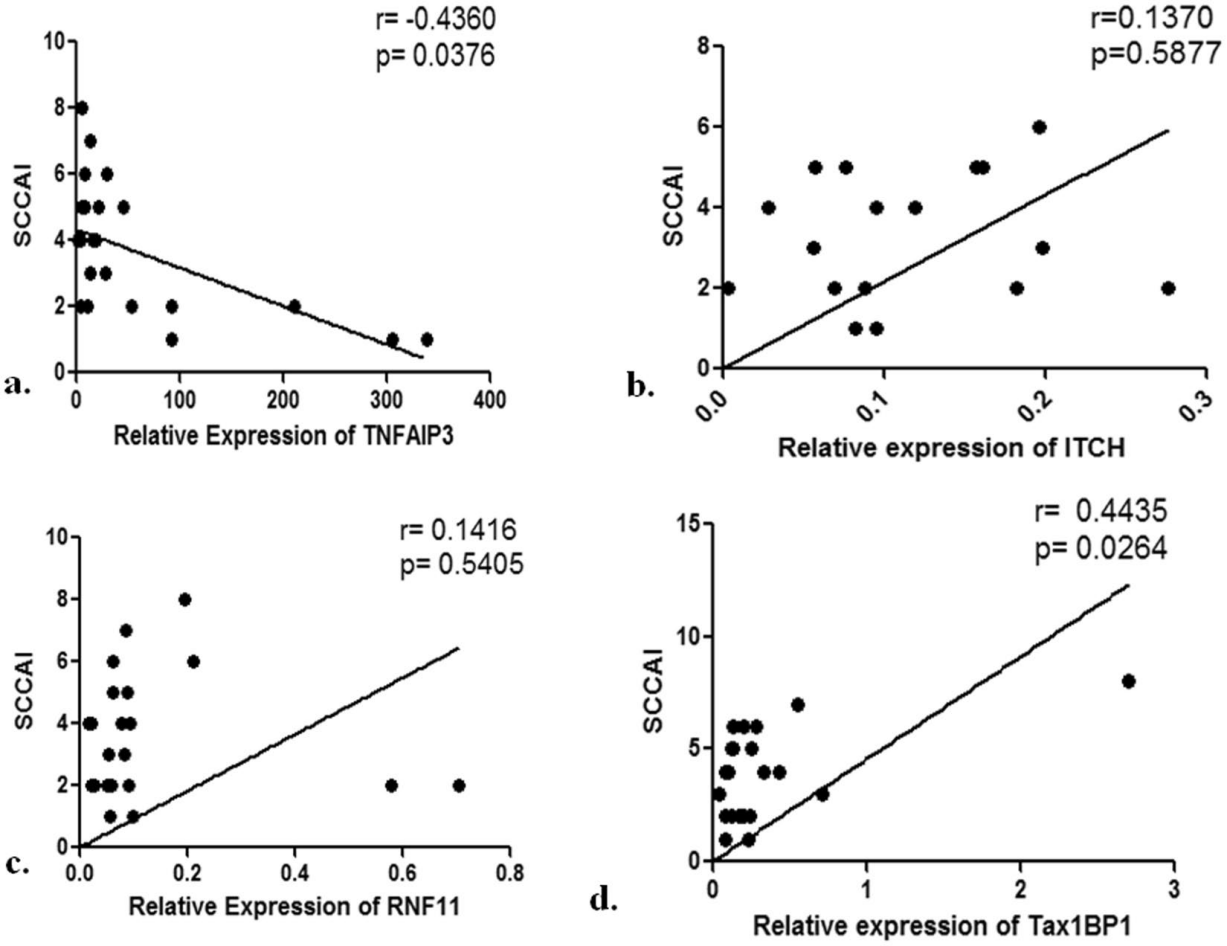
Correlation of mRNA expression of ubiquitin editing complex members with disease activity Correlation of (**a**) TNFAIP3 (**b**) ITCH (**c**) RNF11 and (**d**) Tax1BP1 mRNA expression with disease activity measured by SCCAI score of 1 to 8 in UC patients. r is spearman’s correlation coefficient, p is statistical significance value.

Disease activity was assessed using Simple Clinical Colitis Activity Index (SCCAI) score as referred in Materials and methods. Amongst patients in our study, the SCCAI score ranged between 1 and 8. For patients with SCCAI of 1–8, correlation analysis with TNFAIP3 mRNA levels revealed a significant negative correlation (**r** = **−0**.**4360**, **p** = **0**.**0376**) (Fig. 2a). Among the associated partners of TNFAIP3, ITCH (**r = 0**.**1370**, **p = 0**.**5877**) and RNF11 (**r** = **0**.**1416**, **p** = **0**.**5405**) showed a trend for positive correlation with disease activity (Fig. 2b and 2c). Tax1BP1 on the other hand showed a significant positive correlation with disease activity (**r** = **0**.**4435**, **p** = **0**.**0264**) (Fig. 2d).

### Altered TNFAIP3 mRNA expression is regulated by NFκB

**Figure 3:**
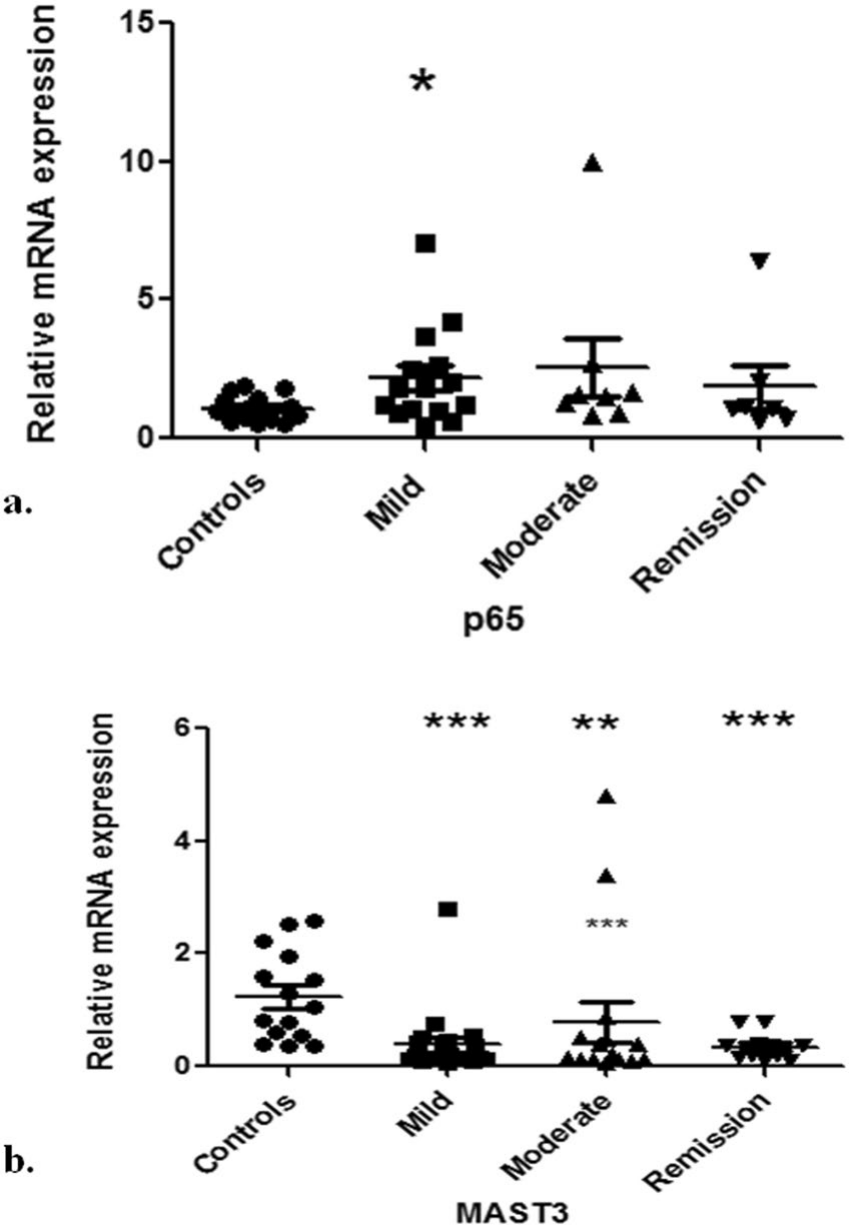
mRNA expression of NFκB p65 subunit and MAST3 in UC Relative mRNA levels of (**a**) p65 (**b**) MAST3 determined by quantitative real time PCR. Controls (n = 15) and UC patients mild (n = 19), moderate (n = 13) and remission (n = 14). GAPDH was used as the reference gene. Data represented as means ± SEM ***p < 0.001 **p < 0.01 *p < 0.05 represents statistical significance of difference in expression with controls.

TNFAIP3 expression is induced by NFκB^31^. Therefore we checked the mRNA expression of p65 subunit of NFκB in UC patients. mRNA expression of p65 increased significantly in UC patients with mild disease activity (Fig. 3a). Its mRNA level was 2.2 fold higher in the mild UC group than in controls (p = 0.03). Amongst the moderate and remission groups, trend for increased p65 mRNA expression was noted. In moderate UC patients, mRNA level of p65 was 2.6 fold higher than the controls (p > 0.05) while in remission patients, the mRNA levels increased by 1.9 fold with respect to controls (p > 0.05). Microtubule associated serine/threonine protein kinase-3 (MAST3) gene is a genetic susceptibility factor for IBD^32^. *In vitro* over expression of MAST3 up-regulates TNFAIP3 expression indicating that it’s expression is modulated by MAST3. Thus, to answer whether MAST3 is also playing a role in increased TNFAIP3 mRNA expression during UC, we determined its mRNA levels. Interestingly, MAST3 mRNA expression showed a significant down-regulation in all disease groups with respect to controls (Fig. 3b). In mild UC patients, its expression decreased to 0.4 fold (p = 0.0001). While in moderate and remission groups, the fold decrease in mRNA expression was 0.8 (p = 0.008) and 0.3 (p = 0.0009) respectively. Mean fold change of mRNA expression of p65 and MAST3 along with p values are given in Table 2.

**Table 2:** mRNA levels of p65 and MAST3.

Gene	Controls	Mild UC	Moderate UC	Remission	Controls vs Mild UC	Controls vs Moderate UC	Controls vs Remission	Mild vs moderate UC	Mild UC vs Remission	Moderate UC vs Remission
	Mean Fold change				P value (Significant p-values are underlined)					
p65	1.1	2.2	2.6	1.9	0.03	0.08	0.53	0.97	0.40	0.34
MAST3	1.2	0.4	0.8	0.3	0.0001	0.008	0.0009	0.61	0.55	0.96

### Protein expression of ubiquitin editing complex members decreases during UC

**Figure 4:**
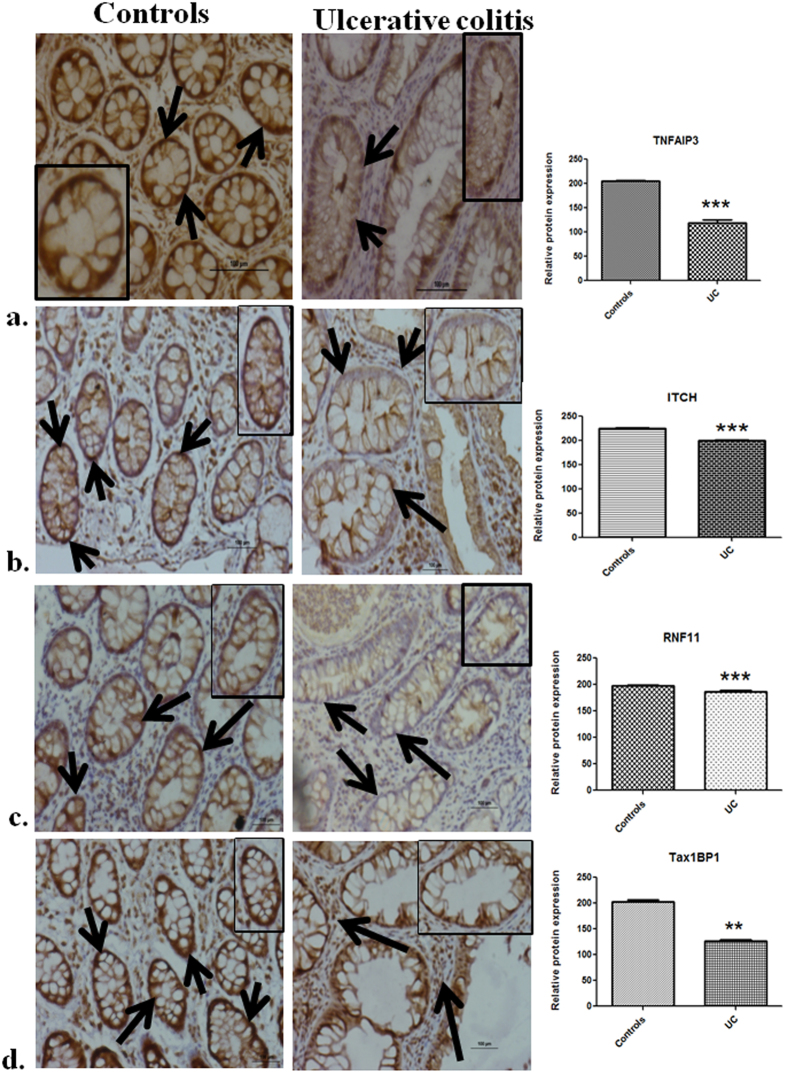
Decreased protein expression of ubiquitin editing complex members in inflamed mucosa of UC patients Detection and evaluation of protein expression of (**a**) TNFAIP3 (**b**) ITCH (**c**) RNF11 and Tax1BP1 in controls and inflamed mucosa of UC patients by immunohistochemistry. Image is representative of three controls and three UC patients. Magnification 20×. Inset shows a single epithelial crypt. Arrows indicate immunoperoxidase staining with DAB (brown in color). Counter staining is seen as blue in color. Data represented as means ± SEM ***p < 0.001 **p < 0.01.

Expression of ubiquitin editing complex members at the protein level was determined by immunoperoxidase staining in colonic biopsies from controls and inflamed areas in UC patients. In case of TNFAIP3, contrary to mRNA expression, protein levels showed down-regulation during UC. TNFAIP3 expression decreased by marked 43% in patients compared to controls (p = 0.0007) (Fig. 4a). TNFAIP3 expression was reduced in both epithelial cells of the crypt and infiltrating cells. Similar to mRNA expression, ITCH, RNF11 and Tax1BP1 protein levels also decreased during UC. ITCH expression was 12% lesser in patients than controls (p = 0.0003) (Fig. 4b). Decrease in ITCH expression was particularly noticeable in the epithelial cells of crypts. RNF11 protein levels decreased upto 6% particularly in the epithelial cells (p = 0.0003) (Fig. 4c). Tax1BP1 expression decreased by 38% in patients compared to controls in both epithelial and infiltrating cells (p = 0.002) (Fig. 4d).

### Reduced levels of ubiquitin editing complex members affects expression of p65 sub-unit of NFκB and NFκB dependent genes

**Figure 5:**
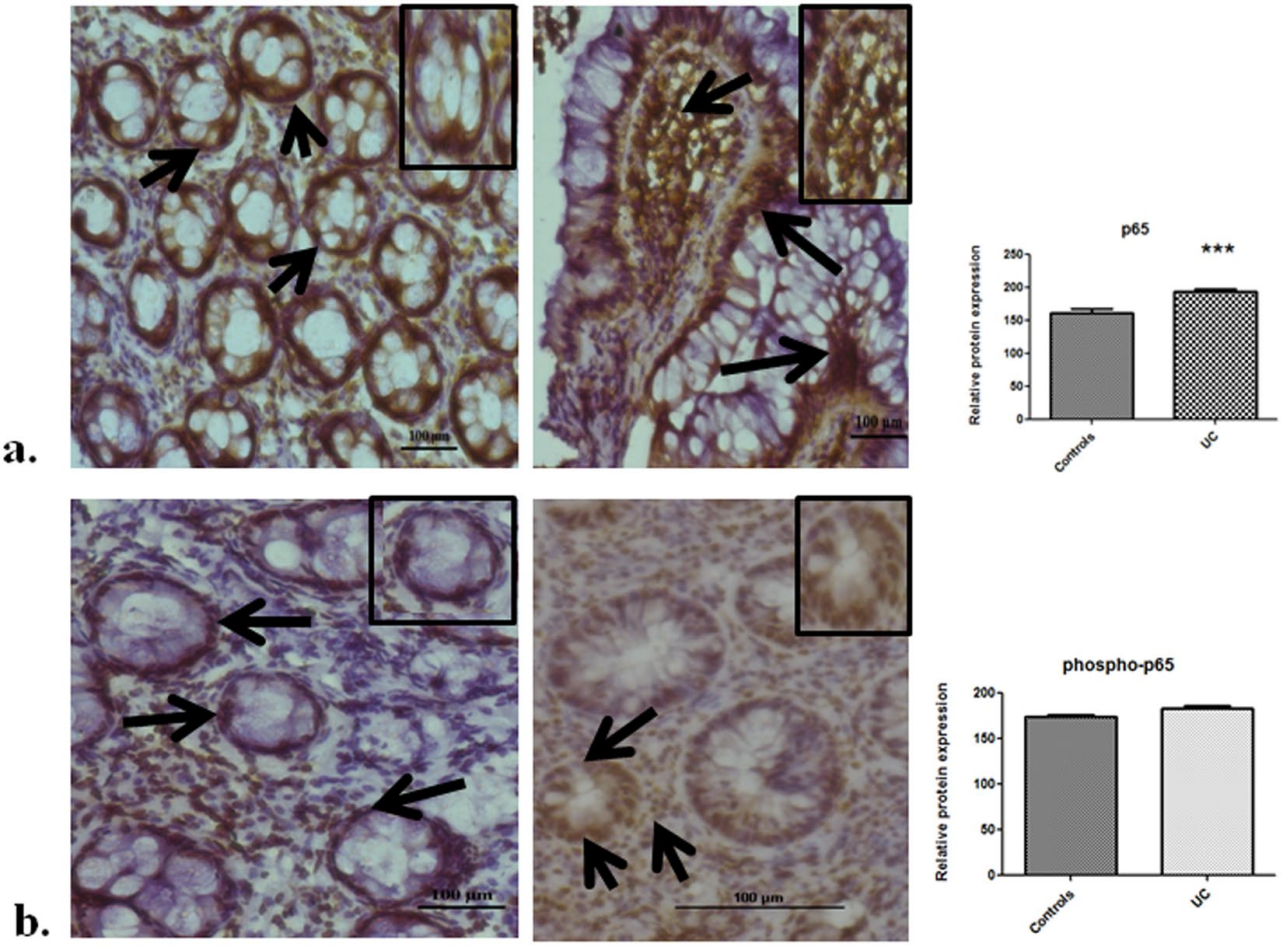
Expression of p65 and phospho-p65 increases during UC Detection and evaluation of protein expression of (**a**) p65 (**b**) phospho-p65 in controls (left panel) and inflamed mucosa of UC patients(right panel) by immunohistochemistry. Image is representative of three controls and three UC patients. Magnification 20X. Inset shows a single epithelial crypt. Arrows indicate immunoperoxidase staining with DAB (brown in color). Counter staining is seen as blue in color. Data represented as means ± SEM ***p < 0.001.

We hypothesized that deficiency of members of ubiquitin editing complex including TNFAIP3 could affect expression of NFκB p65 sub-unit at the protein level. Accordingly, we detected the expression of p65 and its phosphorylated form through immunohistochemistry. A significant increase in protein levels of p65 was observed in inflamed colon tissues of UC patients. As compared to controls, p65 protein expression increased by 21% in patients (p = 0.003) (Fig. 5a). In addition, protein expression of phosphorylated p65 also showed a trend for up-regulation in UC patients. Its expression was 5% greater than the controls (p > 0.05) (Fig. 5b).

**Figure 6:**
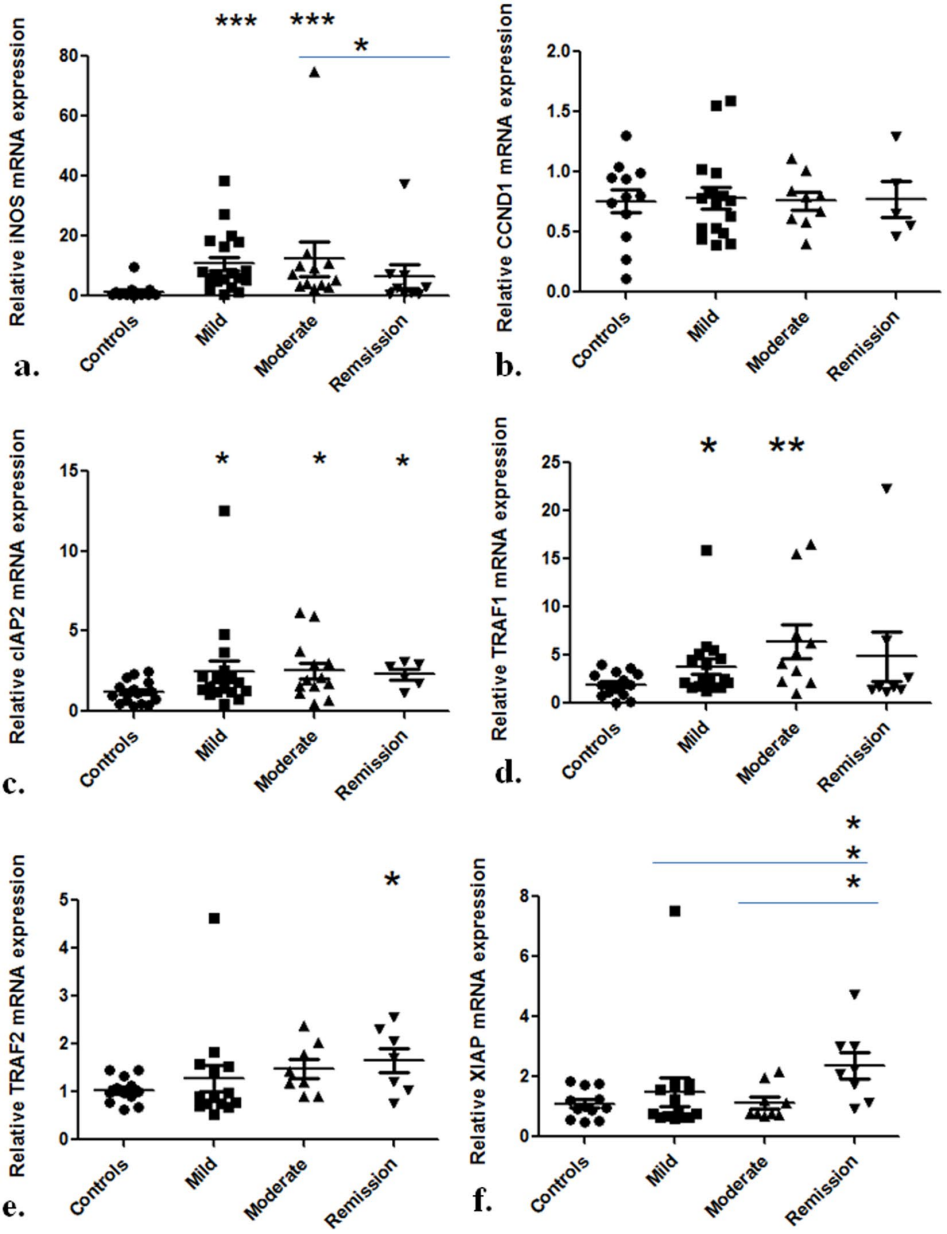
mRNA expression of NFκB regulated genes is up-regulated during UC Relative mRNA levels of (**a**) iNOS (**b**) CCND1 (**c**) cIAP2 (**d**) TRAF1 (**e**) TRAF2 and (**f**). XIAP determined by quantitative real time PCR. Controls (n = 15) and UC patients mild (n = 19), moderate (n = 13) and remission (n = 14). GAPDH was used as the reference gene. Data represented as means ± SEM ***p<0.001 **p < 0.01 *p < 0.05 represents statistical significance of difference in expression with controls. NS = Non-significant.

Further we reasoned that up-regulation of both phosphorylated and unphosphorylated forms of p65 could influence the expression of genes that are regulated at transcriptional level by NFκB. Therefore, we determined expression of six genes downstream of NFκB signaling (Table 3). mRNA levels of the inflammatory marker Inducible nitric oxide synthase (iNOS) were up-regulated during the disease. iNOS mRNA expression was 10.7 fold higher in mild UC patients as compared to controls (p < 0.0001) (Fig. 6a). In patients with moderate UC, it’s expression increased by 12.3 fold with respect to controls (p < 0.0001). iNOS mRNA levels were also elevated in remission patients by 6.5 fold compared to controls (p = 0.12). Further, iNOS fold change in remission patients was significantly lesser than that of moderate UC patients (p = 0.04). We also examined the mRNA expression of cyclin D1 gene (CCND1) that codes for cyclin D1 and plays an important role in cell cycle progression. mRNA levels of CCND1 showed a trend for increased expression in all disease groups of UC (p > 0.05) with respect to controls (Fig. 6b) (Table 3). Next we determined the expression of anti-apoptotic genes i.e. cellular inhibitor-of-apoptosis proteins (cIAP2), Tumor necrosis factor receptor-associated factor 1 (TRAF1), Tumor necrosis factor receptor-associated factor 2 (TRAF2) and X-chromosome-linked inhibitor of apoptosis protein (XIAP). cIAP2 mRNA expression increased during UC (Fig. 6c). In both the mild and moderate UC groups, mRNA levels of cIAP2 were 2.5 fold higher than controls (p = 0.04 and p = 0.02 respectively). While in remission patients, it’s mRNA expression increased by 2.3 fold (p = 0.02) with respect to controls. TRAF1 mRNA levels were 3.8 fold higher in mild UC patients than controls (p = 0.04). While moderate UC group showed an even higher increase in mRNA levels than control group (Fig. 6d). In moderate UC patients, TRAF1 expression increased by 6.4 fold with respect to controls (p = 0.006). mRNA levels of TRAF1 were 4.8 fold higher in remission patients than controls (p > 0.05). TRAF2 mRNA expression increased in mild and moderate UC groups by 1.3 and 1.5 fold respectively (both p > 0.05). On the other hand, in remission patients, its mRNA levels were 1.7 fold higher than controls (p = 0.04) (Fig. 6e). Similarly, XIAP mRNA expression was also higher in mild and moderate UC patients by 1.5 and 1.2 fold respectively (both p > 0.05). While in remission patients, XIAP mRNA levels were 2.4 fold greater than controls (p = 0.01). Fold change of mRNA expression in remission patients was significantly higher than mild and moderate UC patients as well (p = 0.02 and p = 0.01 respectively).

**Table 3:** mRNA levels of NFκB regulated genes.

Gene	Controls	Mild UC	Moderate UC	Remission	Controls vs Mild UC	Controls vs Moderate UC	Controls vs Remission	Mild vs moderate UC	Mild UC vs Remission	Moderate UC vs Remission
	Mean Fold change				P value (Significant p-values are underlined)					
iNOS	1.4	10.7	12.3	6.5	<0.0001	<0.0001	0.12	0.73	0.07	0.04
CCND1	0.7	0.8	0.8	0.8	0.8	0.92	0.87	0.67	0.90	0.90
cIAP2	1.2	2.5	2.5	2.3	0.04	0.02	0.02	0.50	0.31	0.69
TRAF1	2.0	3.8	6.4	4.8	0.04	0.006	0.72	0.15	0.37	0.20
TRAF2	1.0	1.3	1.5	1.7	0.86	0.1	0.04	0.10	0.09	0.60
XIAP	1.1	1.5	1.2	2.4	0.98	0.97	0.01	0.68	0.02	0.01

## Discussion

In this study, we showed that expression of ubiquitin editing complex members was altered in inflamed colon tissues and genes involved in inflammation and inhibiting apoptosis were up-regulated. Hyper-activated NFκB is one of the main factors causing inflammation during UC^6^. This led us to question the role of TNFAIP3, the feedback inhibitor of NFκB activation and its associated partners of the “ubiquitin editing complex”, during inflammation in UC.

Altered expression of TNFAIP3 has been previously reported in other inflammatory disorders like CD, psoriasis, cystic fibrosis and pediatric IBD^30, 33–35^. Though, the nature of alteration may be disease specific. For example in psoriasis, TNFAIP3 mRNA expression increases during the disease. However, its expression decreases during CD, cystic fibrosis and pediatric IBD. In our study, we report significant up-regulated expression of TNFAIP3 mRNA in UC patients as compared to controls. This is consistent with a previous finding that TNFAIP3 mRNA expression increases in inflamed tissues compared to non-inflamed tissues during UC^36^. This suggests that alteration in TNFAIP3 expression is an inherent feature of inflammation during UC. The up-regulation in expression may be due to increased levels of various pro-inflammatory cytokines in inflamed colonic epithelium that activate NFκB^37^. NFκB binds to the two κB binding sites present in the TNFAIP3 promoter that enhances transcription greater than the basal transcription rates leading to its increased levels^38^. Indeed, the mRNA expression of NFκB p65 subunit was up-regulated in our study. Thus, suggesting the role of NFκB in inducing TNFAIP3 expression. MAST3 positively regulates TNFAIP3 expression through the NFκB pathway *in vitro* ^36^. However, our study shows a significant decrease in MAST3 mRNA expression during UC. This is consistent with a previous study where MAST3 expression has been reported to decrease in non-inflamed UC compared to controls^39^. Thus, it indicates that regulation by MAST3 does not contribute to increased TNFAIP3 expression during UC.

We observed a tendency for reduced TNFAIP3 mRNA expression in moderate UC patients as compared to mild UC group. This indicates that there is loss of up-regulation of TNFAIP3 expression during greater disease severity of UC. Thus TNFAIP3 expression may play a role in pathogenesis of disease severity in UC. To confirm this, we carried out correlation analysis of TNFAIP3 mRNA expression and SCCAI score. SCCAI score measures the disease activity and is assigned by the clinician as described in materials and methods section. We found a significant negative correlation between the two parameters. Therefore, as disease activity increases, TNFAIP3 expression decreases. Thus it is likely that reduced TNFAIP3 expression contributes to disease severity of UC. Insufficient up-regulation of TNFAIP3 has also been reported in psoriasis where a significant loss of up-regulation in severe patients compared to mild patients has been observed^33^. Deficiency of TNFAIP3 expression in moderate UC patients may be due to increased degradation of mRNA or reduced transcription rates. Literature reports suggest the latter as the most likely mechanism for TNFAIP3 deficiency during higher disease severity. Epigenetic mechanisms including promoter methylation or polymorphisms in the 5′ region can lead to loss of TNFAIP3 expression^40^. Indeed it has been seen that single nucleotide polymorphisms (SNPs) in intergenic and conserved regulatory regions suppress transcription in rheumatoid arthritis and cause genotype dependent decrease in expression during systemic lupus erythematosus respectively^41, 42^. Further investigation on UC associated TNFAIP3 polymorphisms and their effect on its expression is required.

We also found that altered mRNA expression of TNFAIP3 was accompanied by significant decrease in its protein expression during UC. This may be due to post transcriptional mechanisms like microRNA (miRNA) mediated regulation. miRNAs are small 22 nucleotides long RNA that regulate expression of various genes by repressing translation or degrading target mRNAs. Interestingly, miRNAs are themselves dysregulated in various inflammatory disorders like UC. Indeed one of the miRNAs targeting TNFAIP3 i.e. miRNA 125b is up-regulated during UC^43, 44^. Therefore, it may be a probable mechanism by which TNFAIP3 protein expression is down-regulated during UC. Presence of missense SNPs has been previously reported to decrease protein stability and hence increase protein degradation^45^. Various SNPs have been reported in TNFAIP3 that are associated with different inflammatory disorders^46, 47^. Thus, SNPs in TNFAIP3 can be another means for decreased protein levels. However, this is currently speculative with more studies required to explore presence of UC associated missense SNPs in TNFAIP3 and evaluate their effect on its stability. Reduced TNFAIP3 protein expression is likely to affect intestinal barrier function and thereby increase microbial translocation from the lumen to the intestinal epithelium leading to an enhanced pro-inflammatory immune response and inflammation^28^.

In contrast to TNFAIP3, mRNA and protein expression of partners of TNFAIP3 in ubiquitin editing complex, ITCH, RNF11 and Tax1BP1 was down-regulated during UC compared to controls. ITCH is an E3 ubiquitin ligase thereby, it regulates protein function in important cellular processes such as cell trafficking. Since it is expressed strongly in the gastrointestinal tract, its deficiency as observed by us is likely to affect multiple pathways in the colon including TNFAIP3 mediated NFκB inhibition^48^. RNF11 binds to multiple proteins that are involved in many cellular pathways and mechanisms. In our study we report significant decrease in RNF11 expression in UC patients as compared to controls. In other inflammatory disorders like colon cancer, it has been shown to over express moderately^25^. Further studies are required to know the mechanisms for altered RNF11 expression and its consequences. Studies regarding effect of altered Tax1BP1 expression during inflammatory disorders are currently lacking, but the down-regulated expression observed here is likely to disrupt its function as an ubiquitin adaptor for TNFAIP3 in NFκB inhibition pathway. It can be concluded from our observations that during disease condition, reduced expression of ubiquitin editing complex members is likely to hamper feedback regulation of NFκB activation by TNFAIP3. We also observed that ITCH and RNF11 showed a tendency for positive correlation with disease activity of UC as revealed by correlation analysis. Further, we found that Tax1BP1 showed significant positive correlation with disease activity during UC. However, its implication on disease progression and pathogenesis is yet to be understood. We hypothesized that decreased expression of members of the ubiquitin editing complex could result in increased expression of NFκB. Indeed, we observed that protein expression of NFκB p65 sub-unit was significantly increased in UC patients as compared to controls. This is in consistence with a study carried out by Schreiber *et al*., where p65 expression was reported to increase during UC^6^. Additionally expression of phosphorylated p65 protein also tended to increase during UC in our study.

To further understand the implication of deficiency of ubiquitin editing complex members during inflammation in UC, we looked into the expression of some of the genes that are transcriptionally regulated by NFκB. iNOS is an NFκB inducible gene that synthesizes nitric oxide (NO), a pleiotropic signaling and regulatory molecule that plays an important role in immune response. iNOS codes for the isozyme that produces large amounts of NO for a longer period of time. Increased expression of iNOS is reported to correlate with prolonged inflammation in colon epithelium^49^. This is corroborated by our study as we found a significant increase in iNOS mRNA expression during UC. Excess NO production due to up-regulated iNOS exacerbates pathological features of UC by causing cytotoxicity, activating neutrophils and increased production of nitrosamines that can cause cancer. Further, increased expression of iNOS is a prognostic marker in CRC^50^. The G1/S checkpoint is crucial in cell cycle control. Loss of control at this checkpoint plays a key role in carcinogenesis. Cyclin D1 mediates phosphorylation of retinoblastoma protein (pRb) that is required for passage through this checkpoint. CCND1 codes for the cyclin D1 protein. NFκB regulates cell cycle progression during G_1_ phase by targeting levels of cyclin D1 expression. Previously, increased immunostaining of cyclin D1 in active UC and 36% of UC related CRCs has been reported^51^. We also found a trend for increased CCND1 mRNA expression during UC. Though cyclin D1 protein over expression is more common in sporadic CRCs than UC-associated CRCs, alteration in CCND1 gene expression may be early stage changes in carcinogenesis related with UC.

TNFα and other cytokines released in the colonic epithelium during the local inflammatory response in UC induce apoptosis. NFκB activates certain genes as cyto-protective measure to inhibit apoptosis and therefore prevent epithelial cell loss. Expression of TRAF1, TRAF2, and cIAP2 are known to regulated by NFκB transcriptional activity^52^. In this study, we report significant increase in cIAP2 mRNA levels in inflamed colonic mucosa of UC patients. cIAP2 has been previously reported to be up-regulated in regenerating colonocytes during UC^53^. In addition, cIAP2 is a potent promoter of colitis associated cancer and is frequently over expressed in CRC^54^. TRAF1 and TRAF2 have been previously shown to be present in higher amounts in inflamed colonic mucosa of UC patients than non-inflamed mucosa^55^. This is in agreement with our results where we have also found a significant up-regulation in their expression in UC patients. TRAF1 expression is also induced in human colon cancer cells^56^. XIAP is another anti-apoptotic gene whose expression is regulated by NFκB. XIAP deficiency leads to inherited IBD^57^. However there are no reports regarding its expression in UC patients. In our study we report for the first time that XIAP mRNA expression increases during inflammation in UC. XIAP up-regulation has been previously shown to correlate with colorectal cancer tumor progression^58^.

Therefore we hypothesize that decreased expression of TNFAIP3 along with its partner molecules ITCH, RNF11 and Tax1BP1 leads to uncontrolled activation of NFκB. Since NFκB controls transcription of a variety of genes, many of which include pro-inflammatory ones like iNOS, cell cycle progression (CCND1), inhibitors of apoptosis (cIAP2, TRAF1, TRAF2 and XIAP), their expression in turn is also affected. In our study we have found increase in mRNA expression of these genes. Literature evidence suggests that they play a crucial role in colorectal cancer development. Though the precise mechanism of progression from longstanding UC to colorectal cancer is complex, changes in expression of these genes along with persistent NFκB activation may be the early events in this progression. Deficiency of TNFAIP3 expression thus may be one of the links between prolonged UC and CRC.

## Materials And Methods

### Study subjects

In this study forty six patients between 19 and 65 years of age diagnosed and histologically confirmed as UC cases undergoing colonoscopy at department of gastroenterology, All India Institute of Medical Sciences, New Delhi were recruited. Diagnosis of patients was made on the basis of clinical criteria according to ECCO guidelines^59^. Fifteen individuals devoid of gastrointestinal or liver diseases, undergoing colonoscopic examination for intestinal symptoms like hemorrhoids and dyspepsia and with histologically normal findings constituted the healthy controls (referred to as controls) group. Table 4 lists the demographic and clinical characteristics of controls and UC patients involved in the study. Colonic mucosal biopsies were collected from active UC patients in mild, moderate and remission phases of the disease. Biopsies from severe UC patients were excluded due to overt bleeding in the intestinal mucosa on contact with biopsy forceps. UC activity in patients was classified according to SCCAI score based on clinical characteristics^60^. Disease activity was defined as remission with SCCAI scores below 3. Mild and moderate disease activities were defined as SCCAI scores between 3–6 and 6–11 respectively. Disease extent in patients was identified according to the Montreal classification^61^. Patients were classified as proctitis (E1, only rectal inflammation), left-sided colitis (E2, inflammation limited before the splenic flexure) and pancolitis (E3, inflammation beyond the splenic flexure). All patients were on 5-ASA and azathioprine treatment and were not administered steroids or biological treatments.

**Table 4:** Demographic and clinical characteristics of individuals in the study.

Characteristic	UC patients	Controls
Number	46	15
Gender (Male/female)	21/25	11/4
Age [Mean ± SD] (range in yrs)	40 ± 13.77 (19–65)	38.1 ± 8.82 (26–50)
Disease Duration [Mean ± SD] (range in yrs)	7.70 ± 5.50 (1.17–20)	—
Disease Extent (n/ %)		—
Proctitis	20/43.48%	
Left-sided colitis	15/32.61%	
Pancolitis	11/23.91%	
Disease Severity (n/%)		—
Mild	19/41.3%	
Moderate	13/28.26%	
Remission	14/30.44%	
Treatment (n/%)		—
5-ASA	41/89.13%	
Azathioprine	5/10.87%	
Extra-intestinal manifestation (n/%)		—
Yes	5/10.87%	
No	41/89.13%	
Smoking History (n/%)		
Yes	2/4.35%	0/0%
No	44/95.65%	15/100%
Appendectomy (n/%)		
Yes	0/0%	1/6.67%
No	46/100%	14/93.33%

### Ethical considerations

Ethical approval for this study was obtained from ethics committee, AIIMS (**IEC**/**NP**-**501**/**2013**) and Institute Ethics Review Board, JNU (**IERB**/**349**). All experiments were performed according to relevant guidelines and regulations. Informed consent was obtained from patients and control subjects in written. Demographic and clinical characteristics of individuals in study were obtained through review of medical records and questionnaire response.

### Sample collection

Mucosal colonic pinch biopsy samples were collected in RNA Later (Sigma-Aldrich, St. Louis, MO, USA) for RNA isolation and stored at −20 °C until processing was done. For immunohistochemistry, the biopsy samples were collected in 10% Neutral Buffered Formalin. Samples for both RNA isolation and immunohistochemistry were collected in 1.5 ml Eppendorf tubes containing RNA Later and 10% Neutral Buffered Formalin respectively.

### RNA isolation

30 mg of biopsy tissues were homogenized in a glass hand-held homogenizer. RNA was isolated from homogenized biopsy samples using Total RNA Isolation Kit according to manufacturer’s instructions (Agilent, Santa Clara, CA, USA). Isolated RNA was assessed by Nanodrop 2000 spectrophotometer (Thermo Scientific, Waltham, MA, USA) and electrophoresis for RNA integrity, quality and concentration. RNA samples having A260/280 ratios above 1.8 and showing intact 28S and 18S rRNA bands on 1.5% agarose gel were chosen for subsequent experiments. Samples were stored at −80 °C to prevent degradation. cDNA for Quantitative real time PCR was reverse transcribed from 800ng RNA using Revert Aid reverse transcriptase (Thermo Scientific, Waltham, MA, USA).

### Quantitative Real Time PCR

Quantitative real time PCR was carried out with USB VeriQuest Sybr Green qPCR Master Mix (Affymetrix, Santa Clara, CA, USA). GAPDH was used as internal control for relative quantification using the 2^−ΔΔct^ method^62^. Primers used for amplification of target genes were designed from different exons or from exon-exon boundaries to prevent contamination by genomic DNA. Primer sequences for different genes are given in Table 5. A 20 μL reaction mixture containing 1 μl cDNA, forward and reverse primers (4picomole/μl), 10 μl USB VeriQuest Sybr Green qPCR and 7 μL MilliQ was subjected to real time PCR using the conditions: initial denaturation −94 °C for 2 min followed by 40 cycles of 94 °C for 30 sec and 60 °C for 1 min. The reaction mixtures were added in 96 well optical plates (Thermo Scientific, Waltham, MA, USA), sealed with optical adhesive films (Thermo Scientific, Waltham, MA,USA) and subjected to quantitative PCR in Applied Biosystems 7500 Fast Real Time PCR system (Thermo Scientific, Waltham, MA,USA).

**Table 5:** Primers used in Quantitative Real Time PCR.

Gene	Primer Sequence (5′-3′)	Amplicon size	Reference
TNFAIP3	Forward: GGCGTTCAGGACACAGAC	155 bp	Labbe *et al*.^36^
	Reverse: TTCCAGTTCCGAGTATCATAGC		
ITCH	Forward:AAGGAGCAATGCAGCAGTTTAAC	137 bp	This study
	Reverse: TCTGCCATTGCTGTCTGTTC		
RNF11	Forward: TCTCCCTGCTTCACGAGTCTC	144 bp	This study
	Reverse: GAGTTGCTAGCCGAGTCTGG		
Tax1BP1	Forward: ACAAAGGCCACCTGTCAGAG	132 bp	This study
	Reverse: GGCACATTCTCATCTTCTTTGC		
MAST3	Forward: GCAGCGAAGTGG ACTATGG	134 bp	Labbe *et al*.^36^
	Reverse: GATGGTATTCAGGAGAGATGGG		
p65	Forward: GCCTGTCCTTTCTCATCCCA	87 bp	Huang *et al*.^64^
	Reverse: CTGCCAGAGTTTCGGTTCAC		
iNOS	Forward: CAGCGGGATGACTTTCCAAG	75 bp	Guo *et al*.^65^
	Reverse: AGGCAAGATTTGGACCTGCA		
CCND1	Forward: ACAAACAGATCATCCGCAAACAC	144 bp	Suzuki *et al*.^66^
	Reverse: TGTTGGGGCTCCTCAGGTTC		
cIAP2	Forward: ACACATGCAGCCCGCTTTA	157 bp	Berezovskaya *et al*.^67^
	Reverse: CTCCAGATTCCCAACACCTGA		
TRAF1	Forward: AGAACCCGAGGAATGGCGA	162 bp	Qiao *et al*.^55^
	Reverse: TGAAGGAGCAGCCGACACC		
TRAF2	Forward: AGGTCTGCCCCAAGTTCCC	159 bp	Qiao *et al*.^55^
	Reverse: GCTGTTTCTCACCCTCTACCGT		
XIAP	Forward: GGCAGATTATGAAGCACGGATC	151 bp	Berezovskaya *et al*.^67^
	Reverse: GGCTTCCAATCAGTTAGCCCTC		
GAPDH	Forward: GCTCCTCCTGTTCGACAGTCA	180 bp	Verma *et al*.^68^
	Reverse: GCAACAATATCCACTTTACCAG		

### Immunohistochemistry

Mucosal colonic biopsy samples from three controls and three UC patients fixed in 10% Neutral buffered formalin were paraffin embedded. Sections obtained of uniform sizes (~5 μm) were rehydrated using xylene and ethanol series. Samples were deparaffinized and antigens were retrieved by Heat Induced Epitope Retreival [HIER]. HIER was performed in polypropylene Coplin staining jar placed in a water bath at 92–95 °C. Endogenous peroxidase activity was blocked with 4% hydrogen peroxide and washed thoroughly with PBS. Slides blocked with 10% normal goat serum (Thermo-Fisher Scientific, IL, USA) were incubated with primary rabbit 1:50 anti-human A20 (Sigma, St. Louis, MO, USA), 1:200 anti-human ITCH antibody (Sigma, St. Louis, MO, USA), 1:100 anti- human RNF11 (Thermo-Fisher Scientific, IL, USA), 1:200 anti-human Tax1BP1 (Thermo-Fisher Scientific, IL, USA), 1:100 anti-human p65 (Santa Cruz Biotechnology, Dallas, TX, USA), 1:100 anti-human phospho-NFκB p65 (Ser 276) (Santa Cruz Biotechnology, Dallas, TX, USA) at 4 °C for overnight. The slides were washed thrice with Tris buffered saline (pH 7.5) for five minutes each, after incubation with primary antibody and then incubated with peroxidase labeled anti-rabbit 1:100 secondary antibody (Sigma, St. Louis, MO, USA). The peroxidase substrate 3′-diaminobenzidine (DAB) solution was added to the slides at room temperature and washed off after five minutes. Counterstaining was done with hematoxylin. Lastly, the slides were mounted with D.P.X (Sigma-Aldrich, St. Louis, MO, USA) solution for visualization.

Images were acquired using Nikon Eclipse Ti-S microscope at 20X magnification. Image analysis was performed using NIS-Elements Version 3.0. Protein expression was quantified by reciprocal intensity method as described by Nguyen *et al*.^63^. A minimum of five fields were evaluated for each control and patient slide for statistical validation.

### Statistical analysis

Statistical analysis was carried out using Graph Pad Prism version 5. Difference in mRNA expression between groups was statistically analyzed using Mann-Whitney test. A p value of <0.05 was considered statistically significant. Data is represented as mean ± SEM. Correlation analyses was performed using Spearman rank test.
